# Factors Associated with HIV Testing History among Pregnant Women and Their Partners in Georgia: The ANRS 12127 Prenahtest Trial

**DOI:** 10.1155/2014/356090

**Published:** 2014-09-29

**Authors:** Maia Butsashvili, Maia Kajaia, George Kamkamidze, Patrice Tchendjou, Annabel Desgrees du Loû, François Dabis, Joanna Orne-Gliemann

**Affiliations:** ^1^Health Research Union, 8 Nutsubidze Street, 0177 Tbilisi, Georgia; ^2^Laboratoire Epidémiologie, Centre Pasteur du Cameroun, BP 1274, Yaoundé, Cameroon; ^3^Institut de Recherche pour le Développement, CEPED UMR, Université Paris Descartes (Sorbonne Paris Cité)-INED-IRD, 221 boulevard Davout, 75020 Paris, France; ^4^Centre INSERM U897 “Epidémiologie et Biostatistique”, INSERM, 146 rue Léo Saignat, 33076 Bordeaux, France; ^5^Institut de Santé Publique Epidémiologie Développement (ISPED), Université Bordeaux Segalen, 146 rue Léo Saignat, 33076 Bordeaux, France

## Abstract

Despite the benefits of timely diagnosis of HIV infection and the wide availability of VCT services, the acceptance of HIV testing and counseling still remains a challenge in Georgia. The goal of our study was to assess the history of HIV testing and associated factors among pregnant women. The recruitment of study participants took place during routine antenatal care visits at one of the large Maternity Hospitals in Tbilisi, capital of Georgia. A total of 491 pregnant women were included in the sample. More than a third of women (38.5%) reported that they were tested for HIV before the current pregnancy and almost all of them (91.5%) were tested during previous pregnancies. Bivariate analysis revealed statistically significant association of women's history of HIV testing with age, education level, remunerated activity, history of STI, and multiparity. In multivariate analysis, the only independent predictor of being HIV tested was ever being pregnant. In conclusion, HIV testing history among women at reproductive age was poor in Georgia. Women mostly received HIV testing at prenatal centers. Efforts should be made to promote HIV testing in primary care settings, which would increase its acceptability and overall testing rate in the population.

## 1. Introduction

Despite low HIV prevalence (55.1 cases per 100,000 population in 2011) [1], Georgia is considered to be at high risk of rapid spread of HIV infection to the general population due to high prevalence of injection drug use (estimated number of IDUs was around 40,000 in 2009) [2], high burden of sexually transmitted infections (STIs) (incidence of syphilis was 9.49 per 100,000 population in 2010) [3] and blood borne diseases such as hepatitis B and hepatitis C (HBV incidence of 1.67 and HCV incidence of 0.82 per 100,000 population in 2010; HCV prevalence: 6.7% in 2008) [3, 4], and increased migration to neighboring countries, such as Russia and Ukraine, which are now experiencing severe and growing national HIV epidemics. Until recently the major route of HIV transmission in Georgia was injection drug use. In recent years sexual transmission significantly increased and a shift from IDU to heterosexual transmission was observed in 2011 when heterosexual activity represented 47.4% of overall transmission [1].

Early identification of HIV infection is recognized as a critical component in controlling the spread of the epidemic, not only to decrease morbidity and mortality among infected individuals, but also to reduce onward transmission [5, 6]. Multiple studies have shown that many infected individuals start controlling their behaviors likely to transmit infection to sex or needle-sharing partners once they are aware of their positive HIV status [7–11]. As antiretroviral treatment (ART) lowers HIV viral load and thus reduces the risk for transmission to others [12–14], early referral to medical care and early ART could prevent HIV transmission in communities while reducing a person's risk for HIV-related illness and death. In addition, voluntary HIV counseling and testing (VCT) is an important entry point to other HIV/AIDS services, including prevention of mother-to-child transmission (PMTCT), prevention and management of HIV-related illnesses, and social support. Finally, from a human rights perspective, HIV counseling and testing can play a role in addressing stigma and discrimination.

The National HIV/AIDS Prevention and Control Program has been implemented since 1996 in Georgia [1] and VCT services have been made gradually available at antenatal care clinics, blood banks, and dedicated VCT centers throughout the country. Georgia embarked early on the wide-scale implementation of PMTCT services, mainly with the support of Elizabeth Glaser Paediatric AIDS Foundation (EGPAF), covering HIV counseling and testing of pregnant women in all prenatal centers in the capital of the country, Tbilisi, starting in the 2002–2005 period. The coverage of HIV testing among pregnant women was estimated to be 87% in 2010 [15]. However data on the coverage of HIV testing within the general population in Georgia are scarce and need to be taken with caution, as no population-based surveys have been carried out. The latest UN data on the state of the HIV epidemic in Georgia estimated that 31.8/100,000 adult population had received HIV counseling and testing in 2010 [15].

Better understanding of the context in which people are tested and the factors associated with HIV testing in the general population may be useful for further development of explanatory models of health behavior and for targeting and customizing interventions to further increase the uptake of HIV testing and prevent onwards HIV transmission.

The goal of our analysis was to assess the history of HIV testing and associated factors among pregnant women within the Prenahtest ANRS 12127 study.

## 2. Materials and Methods

The Prenahtest study is a multicountry randomized intervention trial, which evaluated the public health impact of prenatal couple-oriented HIV counseling [16]. The trial was conducted in four urban areas: Yaounde (Cameroon), Pune (India), Santo Domingo (Dominican Republic), and Tbilisi (Georgia) [17].

### 2.1. Study Sample

From March to October 2009 the recruitment of study participants took place during routine antenatal care visits at one of the large maternity hospitals in Tbilisi, capital of Georgia. Pregnant women were informed about the project during pretest HIV counseling sessions and asked to participate in the study privately. Interested women were screened for eligibility criteria. The inclusion criteria for the study enrolment were age 15 years or older, attendance to the first antenatal care visit for this pregnancy, currently having a stable sexual partner, neither the woman nor her partner having been tested for HIV during her current gestational period, accepting to be followed up during her pregnancy and postpartum, and being able to give informed consent. The exclusion criteria included woman or partner already tested for HIV during her current pregnancy, partner working out of the predefined study area or being absent for more than six months, and woman being unwilling/unable to provide address/contact information or having a physical and/or mental impairment at the time of recruitment. Eligible women who signed written informed consent forms were enrolled in the trial and tested for HIV as part of routine prenatal care. Standardized quantitative questionnaires were administered to the participating women before posttest HIV counseling, by a trained interviewer, in a private room. This baseline questionnaire covered sociodemographic characteristics, awareness of and experience with HIV infection and STIs, attitudes and behaviours related to HIV prevention, and sexuality and reproduction among women. The study was approved by IRB 00006752 of Maternal and Child Care Union, November 13, 2008.

### 2.2. Statistical Analysis

Collected data were entered into statistical software EpiData and analyzed using SPSS version 16. Basic descriptive statistics (frequencies and percentages) were computed for all key variables. Chi square test was used to determine the association between a history of HIV testing and different predictive factors including sociodemographic characteristics, reproductive experience, HIV, and STI awareness of women. A *P* value of less than 0.05 was considered as statistically significant. Poisson regression was used for multivariable analysis to identify the strongest predictive factors of HIV testing in women. All potential predictors were included in the full model and the least significant factors were dropped one at a time based on the largest *P* values using a downward stepwise procedure.

## 3. Results

### 3.1. Sociodemographic Characteristics

A total of 491 pregnant women were included in the sample. Three forms were excluded as information was incomplete, so data collected from 488 participants were included in the final analysis. Most of the participants (71.3%) were under the age of 30 (median = 26, IQR = 22–30 years). More than 65% received more than 12 years of education. Only 29.9% of participating women had a remunerated activity at the time of interview. Almost all women (95.3%) were Orthodox Christians and most of them (83.8%) were married. The vast majority of study participants (95.1%) cohabitated with their partners and 65.6% of them also lived with family members (either family in law or woman's family members). About half of the partners (54.5%) were 25 to 34 years old (median = 29, IQR = 25–34). 73.6% had completed secondary education and 70.3% had a remunerated activity. Similar to participants most of the partners (95.9%) were Orthodox Christians.

### 3.2. Family Planning

Most of the women (94.5%) were aware of contraceptive methods, but only 3.9% reported that they never attended family planning (FP) consultation. About 12% of participants responded that they have never heard about the availability of FP services. About 38% of women declared that they never used FP method in their lifetime. The most frequently used contraceptive methods were condom, followed by natural (withdrawal and fertility awareness) methods, pills, intrauterine device, and lactation amenorrhea (Table 1).

### 3.3. STIs Awareness and HIV Risk Perception

To the question “have you ever had STI?” about 15% of women responded “yes.” Most of them reported that they were treated for STIs. Among treated women, 84.2% received medical treatment at health centers, 13.2% underwent self-treatment, and 2.6% were not treated at all. Seventy (14.3%) women reported that their partners never had an STI.

The majority of women (61.9%) did not perceive themselves at risk of HIV infection. Among those who saw themselves at risk of HIV (21.9% of the women surveyed), 52.7% thought they might get HIV by blood transfusion, while others (22.3%) stated that sexual contact put them at risk. Less than a third of women (23.6%) thought that their partners were at risk of HIV infection. The majority of these women (47.8%) considered blood transfusion as the main factor that put their partners at risk of HIV infection, followed by sexual transmission (37.4%) and IVDU (3.5%). About 20% of study participants thought that they were at risk of HIV infection from their partners.

### 3.4. Women's History of HIV Testing and Associated Factors

More than a third of women reported that they were tested for HIV before the current pregnancy and almost all of them were tested during previous pregnancies. Among the other reasons for previous HIV testing were professional requirement and self-motivation (Table 1).

Bivariate analysis revealed statistically significant association of women's history of HIV testing with several factors (Table 2). Women over 30 (prevalence ratio [PR] = 2.01; 95% confidence interval [CI]: 1.63–2.48), with higher education (PR = 1.7; 95% CI: 1.29–2.26) and remunerated activity (PR = 1.45; 95% CI: 1.17–1.81), were more likely to be tested previously for HIV than younger, less educated, and unemployed women. Participants who ever received FP counseling reported higher rates of previous HIV testing compared to those who never attended (73.7% versus 37.1%) with a PR of 13.52 (95% CI: 7.92–23.08). Among women who ever used any FP method a higher proportion were tested for HIV than those among those who never used contraceptives (PR = 2.74; 95% CI: 2.17–3.45). Multiparity was strongly associated with previous HIV testing. Previous HIV testing was reported by only 5.4% of women with first pregnancy. Among multiparous women HIV testing rate was fourteen times higher compared to primiparous women (PR = 13.83; 95% CI: 8.26–23.13). Ever having an STI was also significantly associated with HIV testing (Table 2).

In multivariate analysis, the only independent predictor of HIV testing history among women was ever being pregnant (adjusted PR = 1.55; 95% CI: 1.45–1.64) (Table 2).

### 3.5. Reported Partners' HIV Testing

Fifty-nine (12.1%) participating women reported that their partners had ever been tested for HIV. The main reason for partner testing was professional requirement (55.9%), followed by self-motivation (13.6%) and blood donation (6.8%).

## 4. Discussion

This is the first study in Georgia to assess HIV testing history in any given population and the associated factors. Only 38.5% of the pregnant women enrolled in the Prenahtest trial reported previous HIV testing and only 12% of them reported previous HIV testing among their male partners. Among women, the main reasons for previous HIV testing were a previous pregnancy, professional requirement, and a previous STI. Our study suggests that the majority of women tested for HIV were tested as part of their prenatal care. Multiparity was indeed the most significant factor associated with previous HIV testing among women participating in the study. Other studies from industrialized countries also report the previous pregnancy as a factor associated with HIV testing. Herndon et al. found that the strongest predictor of HIV testing in the past year was previous pregnancy among homeless women in Los Angeles County, USA [18]. The testing rate among women reporting a previous pregnancy was 75.3%. This is similar to the data from some Western European countries such as in Germany where in 2007 the prenatal HIV testing rate in an urban area was 76% [19].

This finding suggests that HIV testing is effectively done during routine prenatal care in Georgia, with a high coverage of PMTCT services. However, women who have never been pregnant rarely receive HIV counseling and testing. Moreover, self-motivation was rarely reported as a reason to test (1.1% of women tested). Overall, these data suggest that women tend to be tested when this service is systematically offered to them, as would be during a pregnancy, and do not themselves proactively seek HIV testing. This attitude could be explained by lack of interest in HIV issues, lack of concern, and low HIV risk perception, factors very often associated with the acceptance of HIV testing. According to the qualitative data also collected within the Prenahtest trial, requesting an HIV test would stigmatize unmarried women as having had an “immoral” behavior and, for married women, would suggest they are questioning their partner's infidelity [20].

Our study results also show that the uptake of HIV testing was associated with a history of STIs. According to the study conducted by US Centre for Disease Control HIV testing acceptance was generally higher (>50%) among people at high risk for acquiring or transmitting the infection (e.g., STD patients, pregnant women at high risk) than among low-risk people [21]. In another study conducted in the US to determine HIV testing rates and factors influencing testing among antepartum women in an obstetric practice, those with a partner at risk or reporting a history of STI were more likely to consent to HIV testing compared to those without. Even after controlling for interaction in a multivariate analysis, a history of sexually transmitted infections for the women or their partners remained significantly and independently associated with a decision to undergo HIV testing [22]. The association we observe in our study between HIV testing experience and history of STIs suggests that STI patients are indeed offered HIV testing.

Finally, 3.7% of women participants reported that their previous HIV testing was a professional requirement. In Georgia, there are several work categories still requiring an HIV testing certificate when applying for a position. This comes from Soviet era, when many institutions were requesting HIV test before admission.

One of the limitations of the study is the fact that the HIV testing history was self-reported by women. We did not seek access to their medical records to validate this information. In spite of these classical limits associated with self-reported behaviours, one of the strengths of the study is the fact that the population interviewed is fairly representative of the general female population of childbearing age of Tbilisi.

In conclusion, the overall HIV testing history among women at reproductive age was low in Georgia. Women mostly received HIV testing at prenatal centers and STI clinics. Efforts should be made to promote HIV testing not only in prenatal followup but also in primary care settings, through provider initiated HIV counseling and testing [23], which could contribute to mainstream HIV testing as a routine health care test, and thus increase its acceptability, which could in turn potentially increase overall testing rate in the population. Eventually, it also seems important to use prenatal HIV counseling and testing services as a tool to offer HIV testing not only to women but also to men. A simple counseling intervention (couple-oriented HIV counseling) provided to women during pregnancy [24] was shown to be acceptable [20] and efficient [17] in Georgia to increase the uptake of partner HIV testing.

## Figures and Tables

**Table 1:** HIV awareness, risk perception, testing history and family planning among pregnant women, Tbilisi, Georgia, 2009-2010, ANRS 12127 Prenahtest.

Characteristics	*N*	%
Can HIV be transmitted from mother-to-child?		
Yes	317	65.0
No	23	4.7
Don't know	133	27.3
Do you think you are at risk of HIV?		
Yes, a lot	5	1.0
Yes, a little	107	21.9
No	302	61.9
Don't know	74	15.2
Do you think your partner is at risk of HIV infection?		
Yes, a lot	2	0.4
Yes, a little	113	23.2
No	287	58.8
Don't know	86	17.6
Do you think you are at risk of being infected with HIV by your partner?		
Yes, a lot	1	0.2
Yes, a little	101	20.7
No	290	59.4
Don't know	96	19.7
Mode of transmission that puts woman under the risk of HIV (her perception)		
Sexual	25	22.3
Blood transfusion	59	52.7
Contaminated medical instruments	37	33.0
Don't know	9	8.9
Other	1	0.9
Mode of transmission that puts partner under the risk of HIV (her perception)		
Sexual	43	37.4
Blood transfusion	55	47.8
IV drug use	4	3.5
Contaminated medical instruments	38	33.0
Doesn't know	10	8.7
Other	2	1.7
Ever tested for HIV before current pregnancy		
Yes	188	38.5
No	300	61.5
Main reason to be tested for HIV		
During previous pregnancy	172	91.5
Job requirement	7	3.7
Self motivated	4	2.1
Medical intervention	3	1.6
Blood donation	2	1.1
Ever heard about Family Planning (FP)		
Yes	462	94.7
No	26	5.3
Ever attended FP consultation session		
Yes	19	3.9
No	469	96.1
Ever used FP method		
Yes	186	38.1
No	302	61.9
Type of FP method ever used		
Pill	49	26.3
IUD	37	19.9
Condoms	118	63.4
Natural (withdrawal, fertility awareness)	66	35.5
Lactational amenorrhea	26	14.0
Other	9	4.8

**Table 2:** Factors associated with previous HIV testing among pregnant women, Tbilisi, Georgia, 2009-2010, ANRS 12127 Prenahtest.

Factors	Total, *n* = 488 (100%)	Previously tested for HIV, *n* = 188 (38.5%)	PR and 95% CI	Adjusted PR and 95% CI
Age				
Less than 30 years	348 (71.3)	104 (29.9)	1	1
30 years and older	140 (28.7)	84 (60.0)	2.01 (1.63–2.48)	1.00 (0.94–1.06)
Education level				
Primary education	167 (34.2)	44 (26.3)	1	1
Secondary education and higher	321 (65.8)	144 (44.9)	1.7 (1.29–2.26)	1.02 (0.98–1.05)
Remunerated activity				
Yes	146 (29.9)	72 (49.3)	1.45 (1.17–1.81)	1.01 (0.96–1.06)
No	342 (70.1)	116 (33.9)	1	1
Ever attended FP consultation session				
Yes	19 (3.9)	14 (73.7)	13.52 (7.92–23.08)	0.92 (0.81–1.05)
No	469 (96.1)	17 (37.1)	1	1
Ever used FP method				
Yes	186 (38.1)	118 (63.4)	2.74 (2.17–3.45)	1.00 (0.94–1.06)
No	302 (61.9)	70 (23.2)	1	1
Perception of herself being at risk of HIV				
Yes	112 (23.0)	64 (57.1)	1.84 (1.46–2.32)	1.01 (0.95–1.06)
No	302 (61.9)	94 (31.1)	1	1
Do not know	74 (15.2)	30 (40.5)	1.30 (0.94–1.80)	0.92 (0.86–1.01)
Perception of her partner being at risk of HIV				
Yes	115 (23.6)	66 (57.4)	1.92 (1.51–2.43)	
No	287 (58.8)	86 (30.0)	1	
Do not know	86 (17.6)	36 (41.9)	1.40 (1.03–1.90)	
Perception of herself being at risk of HIV from partner				
Yes	102 (20.9)	56 (54.9)	1.73 (1.36–2.21)	
No	290 (59.4)	92 (31.7)	1	
Do not know	96 (19.7)	40 (41.7)	1.31 (0.98–1.76)	
Ever been pregnant before				
Yes	231 (47.3)	174 (75.3)	13.83 (8.26–23.13)	**1.55 (1.45–1.64)**
No	257 (52.7)	14 (5.4)	1	
Ever had STI				
Yes	76 (15.6)	42 (55.3)	1.60 (1.26–2.03)	1.04 (0.96–1.11)
No	412 (84.4)	146 (35.4)	1	1
